# Medical Reform

**Published:** 1846-03-01

**Authors:** 


### EDITORIAL, MEDICAL INTELLIGENCE, &c.

*Reform.*—In pursuance of the intentions expressed under this caption in our last number, we proceed to indulge some observations on the means and ends of reform in view of the approaching National Convention. The first object, as it seems to us, to be secured, is a permanent *organization.* It is not to be expected that a convention sitting only a few days, can mature and carry into effect a complete system of medical reform. It may settle some important principles, and enter upon incipient measures, but to perfect plans, and carry them into thorough execution, will require time, and successive sessions. A single convention, simply to compare views, and pass resolutions recommending certain improvements, and disproving certain evils and abuses, would amount to nothing. What is wanted is a permanently organized Congress, acting in behalf of the profession of our whole country, empowered by general consent to take into custody the character and interests of the profession. The general regulation of medical education, and of all matters pertaining to the medical profession, should be vested in this congress. Subordinate to this congress, and standing to it somewhat in the same relation as the State legislatures do to the general government of the United States, we would have an association for each State. Members of the latter should be chosen by the profession in different districts after some regular and uniform principle of representation, which should be prescribed by the National, Medical Congress. State Associations might meet annually. The National Congress once in two or three years.

Let this organization be of the profession alone, independent of all legislation, unless it be advisable to obtain some general corporate qualifications. Let it rely for its continuance and effectiveness on the *esprit du corps* of the profession, and the respect and confidence of the public. It can, as we firmly believe, if the profession unite to sustain it, accomplish all that is desired without any peculiar legal privileges, and in the absence of these it will avoid the popular prejudice against monopolies and aristocratic institutions.

If the National Convention do nothing farther than to take steps which will ensure such an organization as we have just sketched, we, for one, shall be satisfied. To draw up a constitution discriminating between the powers to be vested in the national, or reserved for the state associations, and to embrace all the details which the various objects of such an organization involve, is not to be hastily performed. Perhaps at the first session a committee should be appointed to mature a constitution and report at a subsequent convention, and after being sanctioned by the national convention, it should be submitted to the approval and adoption of the profession in the different states respectively.

We are unable to see any prospect of adequate and permanent measures of reform being successfully carried out except by adopting the plan just mentioned. In view of past experience, and the present condition of things, we can perceive no other method by which the profession throughout our country can co-operate to effect an improvement in medical education, to elevate the standard of acquirement, afford encouragement to scientific investigations, stimulate honorable emulation, and provide for an uniformity of requisitions in all parts of the Union. If an effort of this kind fail, we should have no expectation of reform, at least until matters become so bad that the public, at last, are led to see the necessity of affording proper encouragement and protection in consideration of its own interests. This period will probably not soon arrive. But with proper interest and effort on the part of the profession throughout the country, such an attempt need not fail. Let the profession see to it that delegates are appointed, and such delegates as they would desire to legislate on this subject; let the sacrifice necessary to be present at the convention be shared by contributing to defray their expenses; let the subject be discussed at local associations, so that delegates will go prepared to represent, faithfully, the wishes of their constituents—and, for one, we have every confidence that this movement will result in immeasureable good. We will not, and cannot believe but that the medical profession of our country have the ability and disposition to accomplish every thing desirable relating to reform, and that confidence in the actual existence of these requisites for success, and the conviction that the opportunity for their successful exercise now presents itself, are all that is necessary.

The importance which we attach to the foregoing views will sufficiently account for giving to them precedence. Let us now inquire what are some of the important objects and methods of reform which are to be provided for in the institution of a voluntary, representative government such as we have endeavored to shadow forth. These are, of course, involved in the necessity or propriety of such an organization. We shall, therefore, in this and succeeding numbers give the views which have resulted from what reflection we have bestowed upon the subject.

A prime object should be to encourage scientific investigations, and endeavor to develope and cultivate American medical literature. These ends could be attained by instituting prizes, appointing Committees or individuals to perform specific duties, receiving and publishing among its transactions voluntary communications of sterling value. The latter should be subjected to rigid examination by a competent tribunal before being accepted, so that the honor of being recommended for publication should be a reward for successful efforts, and a stimulus to active competition for it. One of the most serious of the evils affecting the character of the profession in our country, is the absence of any encouragement of national medical literature.

Publishers, with business-like caution, prefer to reprint imported works which have passed the ordeal of criticism, rather than risk the uncertainty attending the publication of those whose merits are to be tested. The consequence is, that the greater portion of our medical literature comes to us in the shape of editorial appendages to foreign works. The want of a national literature has begotten a want of confidence in our writers, unless they have attained great distinction as teachers or practitioners. Look into the libraries of our medical men, and see how few are the standard American works compared with those republished from Britain, France and Germany! Are there not members in the profession in this country possessed of sufficient abilities, industry and ambition to contribute more than has hitherto been done toward a medical literature of our own? We believe in the affirmative to this inquiry; and that the secret of the poverty of the literature of the profession lies in the want of encouragement, and the disadvantages of competition with imported publications. We say, then, let a national association if it be formed, take into consideration the best methods of fostering and rewarding with honorable distinctions, scientific investigations, and meritorious efforts of our own countrymen.

Should a permanent organization be effected embracing, among others, the object of which we are speaking, we should hope soon to see a national institution occupying a position like that of the Academy of France, composed of the most able members of the profession, developing the scientific resources of our country, and enabling the profession to be properly represented in the eyes of other countries. The influence of such an institution upon the profession in our country would be incalculably beneficial. It is not to be concealed that mere success as a practitioner does not always afford sufficient stimulus for the best energies of an active intellect, and beyond the local reputation of being a skilful practitioner, (a reputation often acquired by the ignorant empiric) what distinctions does our profession offer to the ambitious aspirant!—nothing, save the position of a teacher, and we leave our readers to judge how much eclat belongs to this position. The consequence is what might be expected, and what is daily observed, that medicine is not chosen as affording an equal field for distinction with the other professions, and many who enter it, leave it for pursuits in which greater eminence is to be gained by less labor and time. Let it not be inferred from these remarks that we would elevate ambitious motives above those of a higher nature, springing from a sense of duty, or from philanthropy. We speak of things as they are. The former, although secondary, are certainly not unworthy, and should be encouraged rather than repressed. And we believe that if more avenues were opened to merited honors in the medical profession in this country, a great amount of talent and effort would be invited to it which now seeks other departments of effort, energies and abilities hitherto dormant would be awakened to vigorous activity, and the character and usefulness of the profession correspondingly advanced.

We should, also, in progress of time, hope to see arise under the auspices of this national institution, a school embracing extensive and minute instruction in all the departments and specialties of medical science, and of its subsidiary branches. This school should afford opportunities for the prosecution of any subject independently of, or in conjunction with others, according to the taste, or ability of the student. To be a professor in this higher school should be the highest post of honor. It should be open to free competition, and the choice made after some method analogous to the French system of *concours*. In this way opportunity and encouragement would be afforded for profound attainments in every division of our science. We might dwell long on this, as on various other points, but our limits compel us to draw our remarks to a close. We are well aware that there is one difficulty in the way of schemes of this magnitude which will not fail to suggest itself to the reader—all this cannot be accomplished without money: zealous effort alone will not suffice. In our country we labor under a disadvantage which we are mortified to confess is not experienced in countries whose forms of government are less liberal in their fundamental principles than ours. Despotic governments are frequently profuse in their provisions for scientific objects. Ours, especially with regard to medical science, has given little occasion to expect munificent assistance. A national organization, such as we have delineated, ought to be liberally endowed by the general government. Would that the Smithsonian bequest might be appropriated to this object! As to what could be expected from this source, or what could be accomplished without it, others can judge better than ourselves. We have confidence in the principle that *what ought to be done can be done*. Let the work be commenced on a scale broad enough to comprise all that is desirable, and let us in our day do what we can to complete it, leaving, at least, an example to incite to farther exertions those who are to follow us!

We reserve remarks on the subject of medical education for our next number.

